# Abscisic Acid Promotes Petal Senescence in Rose by Regulating RcMYB002

**DOI:** 10.3390/antiox15040415

**Published:** 2026-03-26

**Authors:** Aiyin Cui, Yuzheng Deng, Yuanyuan Kong, Yongjie Zhu, Weibiao Liao

**Affiliations:** 1 College of Horticulture, Gansu Agricultural University, 1 Yinmen Village, Anning District, Lanzhou 730070, China; 1073323020425@st.gsau.edu.cn (A.C.); 1073325010155@st.gsau.edu.cn (Y.K.); 1073323120880@st.gsau.edu.cn (Y.Z.); 2 College of Horticulture and Plant Protection, Inner Mongolia Agricultural University, Hohhot 010011, China; dengyz@imau.edu.cn

**Keywords:** abscisic acid, RcMYB002, petal senescence, *Rosa chinensis*, transcription factor

## Abstract

Flower senescence is a key physiological constraint on the ornamental and commercial longevity of cut roses. Although abscisic acid (ABA) is recognized as a promoter of this process, the molecular circuitry through which ABA operates, particularly the specific contributions of MYB transcription factors, remains largely unexplored. In this study, we identify RcMYB002 as a negative regulator of rose flower senescence. Transient overexpression of *RcMYB002* significantly delays senescence, preserves anthocyanin accumulation, and modulates antioxidant enzyme activities in a time-dependent manner, consequently attenuating ABA-triggered oxidative stress. In contrast, silencing *RcMYB002* accelerates senescence-associated phenotypes. At the molecular level, ABA suppresses *RcMYB002* transcript accumulation, while yeast one-hybrid (Y1H) assays indicate that RcMYB002 interacts with the promoter regions of senescence-associated genes *SAG12* and *SAG21*, consistent with a role in their transcriptional regulation. Taken together, our results support a model in which ABA promotes flower senescence by downregulating RcMYB002, thereby derepressing downstream senescence-executing genes. This work provides a molecular basis for understanding flower senescence and offers a potential target for extending rose vase life.

## 1. Introduction

Abscisic acid (ABA) is a pivotal phytohormone that regulates a wide range of physiological processes in plants, including seed dormancy, stomatal closure, and adaptive responses to abiotic stresses such as drought and salinity [1]. ABA is predominantly synthesized via the indirect carotenoid pathway, in which 9-cis-epoxycarotenoid dioxygenase (NCED) serves as the key rate-limiting enzyme [2]. For instance, in peach, two NCED isozymes, *PpNCED1* and *PpNCED5*, show expression patterns that are highly correlated with endogenous ABA levels. Heterologous expression of these genes in a transgenic callus system further confirmed their role in promoting ABA biosynthesis, suggesting that they likely accelerate cellular senescence via reactive oxygen species (ROS)-mediated signaling [3]. Growing evidence indicates that ABA is a robust promoter of senescence in multiple plant organs, including leaves and petals [4,5]. During petal senescence, endogenous ABA levels typically increase, thereby triggering a series of biochemical and molecular events that result in the dismantling of cellular structures and ultimately lead to tissue death [6]. This process is characterized by pigment degradation, loss of membrane integrity, and accumulation of reactive oxygen species (ROS) [6,7,8]. The core ABA signaling pathway, comprising PYR/PYL receptors, PP2C phosphatases, and SnRK2 kinases, transduces hormonal signal through a phosphorylation cascade that activates ABF-type transcription factors [9]. These activated ABFs in turn induce the expression of senescence-associated genes (*SAG*s), thereby executing the senescence program [10]. Upon perception of external stimuli, ABA biosynthesis is rapidly upregulated, and the hormone is recognized by specific receptors, initiating a signaling cascade that ultimately engages downstream transcriptional networks [11,12].

The MYB family is one of the largest and most functionally diverse transcription factor families in plants, originating from the avian myeloblastosis viral oncogene homolog (v-Myb) [13]. In plants, MYB TFs have expanded considerably and play regulatory roles in a multitude of biological processes, including pathogen and pest resistance, nutrient and toxin uptake, drought and salt tolerance, trichome development, stamen formation, leaf senescence, and the biosynthesis of flavonoids and terpenoids [14,15]. In addition to directly regulating target genes involved in phytohormone signaling, ROS homeostasis, and secondary cell wall formation, MYB TFs often serve as critical hubs for cross-talk between different signaling pathways [16]. Several MYB members have been implicated in flower senescence. For example, *RhMYB108*, an R2R3-MYB TF in rose (*Rosa hybrida*), integrates ethylene and jasmonate signals to promote petal senescence by activating downstream NAC-type senescence-associated genes (SAGs) [17]. Similarly, *PlMYB308* in herbaceous peony contributes to ethylene accumulation during petal senescence; its silencing in peony and overexpression in tobacco respectively extended and shortened flower longevity, underscoring its functional conservation [18]. In Arabidopsis, AtMYB44 modulates ABA sensitivity by competing with ABI1 to bind to the ABA receptor RCAR1/PYL9, thereby fine-tuning the core signaling circuitry [19]. Moreover, MYB15 and MYB96 enhance drought tolerance through ABA-mediated stomatal regulation and stress-responsive gene expression [20,21], while MYB37 functions in drought adaptation via ABA-dependent pathways [22]. Despite these advances, the specific roles of MYB-related TFs in ABA-mediated petal senescence, particularly in ornamental species such as rose, remain poorly understood, representing a critical gap in our knowledge of the transcriptional networks that govern floral longevity.

Rose (*R. chinensis*) is one of the most economically important ornamental plants worldwide, valued for its diverse flower colors and forms [23]. However, the postharvest vase life of cut roses is often limited by rapid petal senescence, resulting in substantial economic losses. Although ABA has been established as a promoter of petal senescence in roses, the downstream transcriptional mechanisms, particularly the involvement of MYB-related transcription factors, are not fully elucidated. In this study, we identified RcMYB002, an MYB-related family member in rose, as an ABA-responsive gene predominantly expressed in petal tissues. Integrating physiological analyses and transient genetic manipulation (overexpression and silencing), we demonstrate that RcMYB002 functions as a negative regulator of petal senescence. Furthermore, we provide evidence that ABA promotes petal senescence, at least in part, by suppressing RcMYB002 expression. Our findings reveal a novel ABA-RcMYB002 regulatory module in rose petal senescence, providing new insights into the molecular basis of flower longevity and potential strategies for improving the postharvest quality of cut flowers.

## 2. Materials and Methods

### 2.1. Plant Materials, Growth Conditions and Treatments

Petals from fresh rose (*R. chinensis* ‘Carola’) flowers at opening stage 2 (characterized by concave sepals and loose outer petals) were used as experimental material. The rose plants were grown in an experimental greenhouse at Gansu Agricultural University, Lanzhou, China, under standard controlled conditions with optimized temperature, light, and water supply. The outermost petals were collected, and petal discs (10 mm diameter) were excised using a punch tool. For petal disc assays, each biological replicate consisted of independent rose plants, and at least 30 petal discs were used per biological replicate. For overexpression and virus-induced gene silencing (VIGS) assays, each biological replicate also represented independent plants, and at least 20 petal discs were collected per replicate for further analysis. All data were obtained from three independent biological replicates (*n* = 3). For treatment, petal discs were sprayed with either 50 μM abscisic acid (ABA) or 50 μM fluridone (FLU), an ABA biosynthesis inhibitor. Control discs were placed on filter paper moistened with deionized water, following the method of Shahbani et al. [24]. All petal discs were placed flat on filter paper in petri dishes and incubated under controlled conditions for phenotypic observation. The filter paper was moistened daily with deionized water, and treatment groups were re-sprayed daily with ABA or FLU solutions until petal senescence. ABA and FLU were dissolved in anhydrous ethanol to prepare stock solutions. Working solutions were obtained by diluting the stock solutions with distilled water, and the control solution contained an equivalent concentration of ethanol. Phenotypic changes were recorded throughout the experimental period.

### 2.2. RNA Extraction and RT-qPCR Analysis

Total RNA was extracted from petal samples using TRIzol reagent (Invitrogen, Carlsbad, CA, USA) according to the manufacturer’s protocol [25]. RNA purity and concentration were assessed using a Pultton P100+ ultra-micro spectrophotometers (Wuzhou Dongfang, Beijing, China). Only RNA samples with A260/A280 ratios between 2.0 and 2.1 were used for subsequent analyses. First-strand cDNA was synthesized from 1 μg of total RNA using the FastQuant First Strand cDNA Synthesis Kit (Tianen, Beijing, China), following the manufacturer’s instructions. The reverse transcription reaction was performed under the following conditions: 37 °C for 15 min, 85 °C for 5 s, and final hold at 4 °C. For qRT-PCR, we used a LightCycler 480 Real-Time PCR System (Roche Applied Science, Penzberg, Germany) and SYBR Green Premix Pro Taq HS Premix kit (Hunan Aikori Biotechnology Co., Ltd., Changsha, China).

Quantitative real-time PCR (qRT-PCR) reaction mixture comprised 10 µL of 2× SYBR Green Pro Taq HS Premix, 0.4 µL of forward primer, 0.4 µL of reverse primer, 2 µL of complementary DNA (cDNA), and 7.2 µL of double-distilled water (ddH_2_O). Melt curve analysis was conducted after amplification to verify the specificity of each PCR product, which yielded a single sharp peak with no primer-dimer formation or non-specific amplification. Primer amplification efficiency was determined by standard curve analysis and ranged from 90% to 110%, with correlation coefficients (R^2^) > 0.99 for all primer pairs. The primers employed in the qRT-PCR were designed using Primer Premier 5.0 software (Premier Biosoft Corporation, San Francisco, CA, USA), with *Rcactin* (GenBank accession number AB239794) serving as the internal reference gene. The sequences of all primers are listed in Table S1. The data can be accessed via the following link: https://www.ncbi.nlm.nih.gov/. The 2^−∆∆CT^ calculation method was used to calculate the relative expression of each gene. Each gene’s relative expression was calculated by comparing it with that at 0 h for each treatment. All experiments were performed three times separately [23].

### 2.3. Transient Overexpression Assay

To generate the overexpression construct, the coding sequence of *RcMYB002* lacking the stop codon was amplified and subcloned into the PAC004 vector, resulting in pAC004-*RcMYB002*-HA. Both the recombinant construct and the empty pAC004 vector (negative control) were introduced into *Agrobacterium tumefaciens* strain GV3101 via transformation. Agrobacterial cultures were pelleted and resuspended in infiltration medium containing 10 mM MgCl_2_, 10 mM MES (pH 5.6), and 200 μM acetosyringone (AS), adjusted to a final OD_600_ of approximately 0.8. The suspensions were incubated at room temperature in darkness for 3–4 h prior to infiltration [2]. Vacuum infiltration was performed by submerging whole rose plants and excised petal discs (1 cm diameter) in the bacterial suspension and applying a vacuum of 0.07 MPa. Following gradual vacuum release, the infiltrated materials were rinsed thoroughly with deionized water and maintained in darkness at 8 °C for 3 d. The transcript level of *RcMYB002* in each biological replicate was verified by qRT-PCR to ensure comparable transformation efficiency among different treatments. Only samples with stable and consistent overexpression efficiency were used for further phenotypic analysis. Afterwards, samples were transferred to ambient conditions for phenotypic evaluation. Petal discs were examined daily for senescence-associated changes until necrosis became evident. The experiment comprised three independent biological replicates, all of which exhibited comparable phenotypic outcomes. For gene expression analysis, petal tissues were collected at indicated time points after incubation in deionized water at 23 °C. A complete list of primers used in this study is provided in Table S2.

### 2.4. Virus-Induced Gene Silencing

To achieve targeted silencing of *RcMYB002*, virus-induced gene silencing (VIGS) was performed according to previously described methods [26,27]. *Agrobacterium tumefaciens* strain GV3101 transformed with pTRV1, pTRV2, or pTRV-RcMYB002 was grown to appropriate density, pelleted, and resuspended in infiltration medium containing 10 mM MgCl_2_, 10 mM MES, and 200 μm AS, adjusted to a final optical density at 600 nm (OD_600_) of approximately 0.8. The transcript level of *RcMYB002* in each biological replicate was verified by qRT-PCR to ensure comparable transformation efficiency among different treatments. Only samples with stable and consistent silencing efficiency were used for further phenotypic analysis. For experimental infiltration, bacterial suspensions were combined in a 1:1 (*v*/*v*) ratio of pTRV1 with either pTRV2 (empty vector control) or pTRV-*RcMYB002* [28]. The infiltration procedure and subsequent treatment conditions were identical to those used in the transient overexpression assay. A complete list of oligonucleotide primers employed in this study is provided in Table S2.

### 2.5. Electrolyte Leakage Assay

Electrolyte leakage rates were determined according to the protocol described by [29], with minor modifications. For each treatment, 12 rose petal discs were submerged in 30 mL of 0.4 mM mannitol solution and incubated at ambient temperature under gentle agitation for 3 h. The initial electrical conductivity of the solution was measured using a conductivity meter (ST3100C; Ohaus Corporation, Parsippany, NJ, USA). Subsequently, samples were heated at 85 °C for 20 min to release total electrolytes, and the final conductivity was recorded. The electrolyte leakage rate was expressed as the percentage (or ratio) of initial conductivity relative to total conductivity.

### 2.6. Determination of Anthocyanin and Malondialdehyde (MDA) Content

Petal samples were collected, immediately frozen in liquid nitrogen, and ground to a fine powder. Total anthocyanins were extracted by incubating the powdered tissue in extraction solution (1% HCl in methanol) at room temperature in the dark for 4 h with gentle shaking. The extract was then centrifuged at 8000× *g* for 20 min, and the resulting supernatant was collected and filtered through a 0.2 μm filter. Anthocyanin content was quantified using the pH differential method [30]. The total anthocyanin content was calculated and expressed as cyanidin-3-glucoside equivalents in milligrams per 100 g fresh weight (FW). All analyses were performed with three biological replicates.

Malondialdehyde (MDA) content was determined following the thiobarbituric acid (TBA) method described by [31], with slight modifications. Briefly, 0.5 g of rose petals were homogenized in a pre-cooled mortar with a small volume of 10% trichloroacetic acid (TCA) and an appropriate amount of quartz sand. The homogenate was centrifuged at 12,000× *g* for 15 min at 4 °C. Subsequently, 3 mL of the resulting supernatant was mixed with an equal volume of 10% TCA containing 0.5% (*w*/*v*) TBA. The mixture was incubated in a boiling water bath for 15 min and then immediately cooled on ice. After centrifugation at 12,000× *g* for 15 min at 4 °C, the absorbance of the supernatant was measured at 450 nm, 532 nm, and 600 nm using a spectrophotometer.

### 2.7. Determination of Hydrogen Peroxide (H_2_O_2_) and Superoxide Anion (O_2_^−^) Content

Superoxide anion (O_2_^−^) content was measured according to the method described by Zhang et al. [32], with minor modifications. Frozen petal tissue (1 g) was ground and homogenized in 3 mL of 65 mM potassium phosphate buffer (pH 7.8), followed by centrifugation at 12,000× *g* for 10 min at 4 °C. An aliquot (1 mL) of the resulting supernatant was mixed with 0.9 mL of 65 mM phosphate buffer (pH 7.8) and 0.1 mL of 10 mM hydroxylamine hydrochloride, and the mixture was incubated at 25 °C for 20 min. Subsequently, 1 mL of 17 mM sulfanilamide and 1 mL of 7 mM α-naphthylamine were added, and the reaction was allowed to proceed for an additional 20 min at 25 °C. After incubation, an equal volume of ethyl ether was added, and the mixture was centrifuged at 4000 × *g* for 5 min. The absorbance of the aqueous phase was measured at 530 nm using a spectrophotometer. The O_2_^−^ content was quantified based on a standard curve prepared using known concentrations of sodium nitrite (NaNO_2_).

Hydrogen peroxide (H_2_O_2_) content was determined according to the protocol described by Zhang et al. [32] with minor modifications. Samples (0.2 g) were homogenized in an ice bath with 2 mL of 0.1% (*w*/*v*) trichloroacetic acid (TCA). The homogenate was centrifuged at 3000 rpm for 10 min at 4 °C. An aliquot (0.5 mL) of the supernatant was combined with 0.5 mL of 10 mM potassium phosphate buffer (pH 7.0) and 1 mL of 1 mM potassium iodide (KI). The content of H_2_O_2_ was estimated by measuring the spectrum absorbance of the supernatant at 415 nm and using a standard curve plotted with a known concentration of H_2_O_2_.

### 2.8. Determination of Antioxidant Enzyme Activity

Antioxidant enzyme activities were measured according to established spectrophotometric methods. Rose petal tissue (0.5 g) was ground to a fine powder in liquid nitrogen and homogenized in 10 mL of ice-cold phosphate buffer (50 mM PBS, pH 7.8). The homogenate was centrifuged at 6000× *g* for 20 min at 4 °C, and the resulting supernatant was collected for subsequent enzyme assays.

Superoxide dismutase (SOD) activity was determined using the photochemical NBT reduction method. The reaction mixture contained 50 mM phosphate buffer (pH 7.8), 13 mM methionine, 75 μM NBT, 10 μM EDTA-Na_2_, and 2 μM riboflavin. The reaction was initiated by adding 0.1 mL of enzyme extract to 2.9 mL of the reaction mixture. The tubes were illuminated at 25 °C for 20 min, and the absorbance was measured at 560 nm. SOD activity was calculated based on the inhibition of NBT photoreduction according to the method of Giannopolitis and Ries [33].

Catalase (CAT) activity was assayed by monitoring the decomposition of H_2_O_2_ at 240 nm according to Aebi [34]. The reaction mixture consisted of 50 mM phosphate buffer (pH 7.0) and 30 mM H_2_O_2_. Enzyme extract (0.1 mL) was added to 2.9 mL of reaction mixture, and the decrease in absorbance was recorded at 30-s intervals for 2 min.

Peroxidase (POD) activity was determined using guaiacol as a substrate, following the method of Klapheck et al. [35]. The reaction mixture contained 50 mM phosphate buffer (pH 6.0), 20 mM guaiacol, and 10 mM H_2_O_2_. Enzyme extract (40 μL) was added to 3 mL of reaction mixture, and the increase in absorbance at 470 nm was recorded every 30 s for 2 min.

Ascorbate peroxidase (APX) activity was measured by monitoring the oxidation of ascorbate at 290 nm according to Nakano and Asada [36]. The reaction mixture contained 50 mM phosphate buffer (pH 7.0), 0.5 mM ascorbic acid, 0.1 mM EDTA-Na_2_, and 1.0 mM H_2_O_2_. Enzyme extract (0.1 mL) was added to 2.9 mL of reaction mixture, and the decrease in absorbance was recorded every 30 s for 2 min.

All enzyme activities were expressed as units per gram fresh weight (U g^−1^ FW). Each assay was performed with three biological replicates per sample.

### 2.9. Yeast One-Hybrid Assay

The full-length open reading frame of *RcMYB002* was amplified from rose cDNA and cloned into the pGADT7 prey vector using *Nde* I and *Xho* I restriction sites. The promoters of *RcSAG12* (1050 bp) and *RcSAG21* (849 bp) were separately inserted into the vector via *Kpn* I and *Xho* I digestion. The resulting pBait-AbAi-promoter vector was then linearized using *Bbs* I and introduced into the yeast Y1H strain. Subsequently, the positive plaques were selected on SD/−uracil (Ura). Suitable concentrations of aureobasidin A (AbA) were picked to suppress self-activation. The pGADT7-MYB002 plasmid was then transformed into a positive pBait-AbAi-promoter yeast strain, spotted on SD/−leucine (Leu) medium (±AbA), diluted in a concentration gradient, and cultured for 3 d. A list of primers used for vector construction is provided in Table S3.

### 2.10. Statistical Analysis

Raw data were recorded and processed using Microsoft Excel 2017 (Microsoft Corporation, Redmond, WA, USA). All statistical analyses were conducted using SPSS software (version 22.0, IBM Corporation, Armonk, NY, USA). Results are expressed as mean ± standard error (SE) from three independent biological replicates (*n* = 3). For multi-group comparisons, statistical significance was evaluated by one-way analysis of variance (ANOVA), followed by Duncan’s multiple range post hoc test. A probability level of *p* < 0.05 was considered statistically significant. Pairwise comparisons between two groups were performed using two-tailed Student’s *t*-test, with significance thresholds set at * *p* < 0.05 and ** *p* < 0.01. All graphical representations were generated using GraphPad Prism (version 9.0.0; GraphPad Software, San Diego, CA, USA).

## 3. Results

### 3.1. ABA Accelerates Petal Senescence of Rose Petals

**Figure 1:**
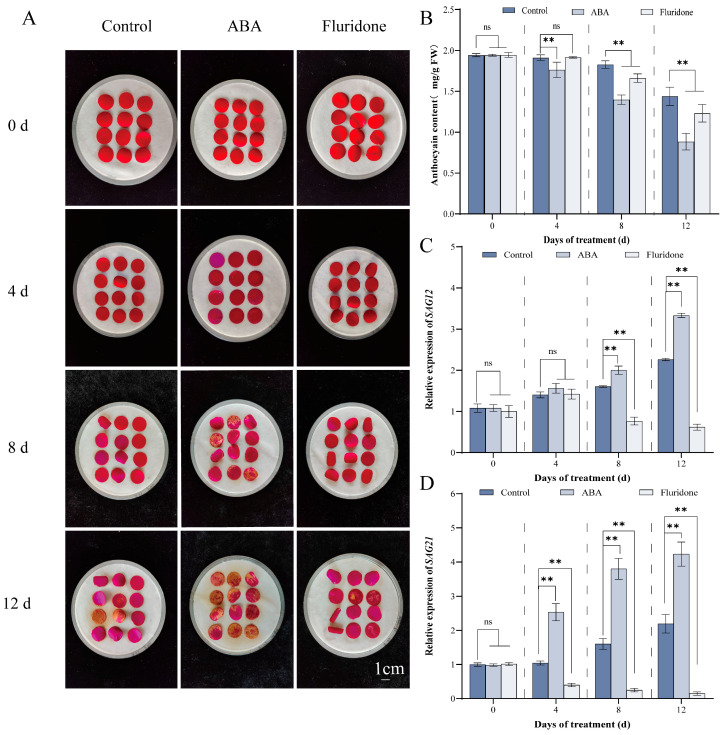
Abscisic acid accelerates senescence in rose petals. (**A**) Representative images showing phenotypic progression of rose petals following treatment with Control (deionized water), ABA (50 μM), or fluridone (50 μM) at different time points. (**B**) Quantification of anthocyanin content in petals subjected to the same treatments. (**C**,**D**) Time-course expression analysis of senescence-associated genes *SAG12* (**C**) and *SAG21* (**D**) in response to Control, ABA, and fluridone treatments, as determined by qRT-PCR. Values represent mean ± SE from three independent biological replicates. Asterisks indicate significant differences between the treatment and Control groups within the same time point ** *p* < 0.01 (Student’s *t*-test); ‘ns’ indicates no significant difference. All experiments were performed with at least three biological replicates.

To elucidate the regulatory function of ABA in petal senescence, we treated rose petals with exogenous ABA and fluridone, a specific inhibitor of ABA biosynthesis. Phenotypic analysis revealed that exogenous ABA markedly accelerated the senescence process (Figure 1A), as evidenced by earlier onset of wilting, fading pigmentation, and inward curling morphological hallmarks of petal senescence [37]. Conversely, inhibition of ABA biosynthesis by fluridone significantly postponed these senescence-associated phenotypic changes, with treated petals retaining turgor and pigmentation for an extended duration (Figure 1A). Given that anthocyanin degradation is a key biochemical event accompanying petal senescence [38], we quantified anthocyanin content over the experimental time course. ABA-treated petals exhibited a more rapid decline in anthocyanin levels, whereas fluridone-treated petals retained relatively higher anthocyanin content throughout the observation period (Figure 1B). Furthermore, quantitative RT-PCR analysis demonstrated that ABA treatment induced the expression of multiple senescence-associated genes (SAGs) at several time points, with both earlier onset and greater magnitude of induction compared to control samples (Figure 1C,D). These results indicate that ABA promotes rose petal senescence by accelerating visible senescence symptoms, pigment degradation, and SAG expression.

### 3.2. ABA Treatment Damages the Cell Membranes of Rose Petals and Disrupts the Redox Balance

**Figure 2:**
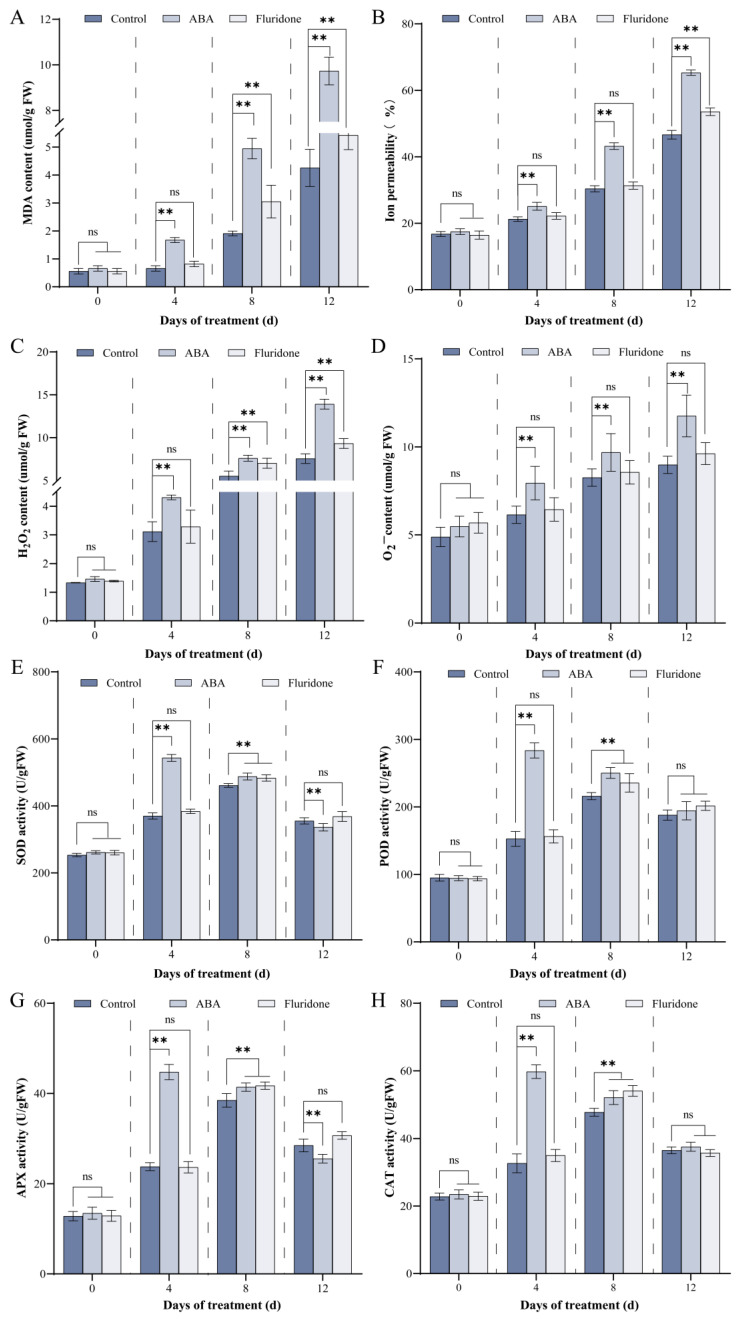
Physiological and biochemical responses of rose petals to ABA treatment. Temporal dynamics of oxidative stress and antioxidant defense parameters in rose petals following treatment with Control (deionized water), ABA (50 μM), or fluridone (50 μM). (**A**) Malondialdehyde (MDA) content, a marker of lipid peroxidation. (**B**) Electrolyte leakage rate, indicating membrane permeability. (**C**) Hydrogen peroxide (H_2_O_2_) content. (**D**) Superoxide anion (O_2_^−^) content. Activities of antioxidant enzymes: (**E**) superoxide dismutase (SOD), (**F**) peroxidase (POD), (**G**) ascorbate peroxidase (APX), and (**H**) catalase (CAT). All measurements were performed at the indicated time points. Values represent mean ± SE from three independent biological replicates (*n* = 3). Asterisks denote statistically significant differences compared with Control as determined by Student’s *t*-test (** *p* < 0.01); ‘ns’ indicates no significant difference.

To further explore the physiological and biochemical effects of ABA on rose petal senescence, we analyzed markers of membrane integrity and oxidative stress. MDA content, an indicator of lipid peroxidation, increased over time in all treatments but was significantly higher in ABA-treated petals compared to the control at most time points. In contrast, fluridone-treated petals exhibited lower MDA levels, indicating reduced membrane damage (Figure 2A). Similarly, electrolyte leakage—a measure of membrane permeability—was markedly elevated in ABA-treated petals (Figure 2B). Consistent with enhanced membrane damage, ABA treatment led to a significant accumulation of reactive oxygen species (ROS). Both H_2_O_2_ and O_2_^−^ contents were substantially higher in ABA-treated petals (Figure 2C,D), whereas fluridone-treated petals maintained lower ROS levels, indicating alleviated oxidative stress. To assess the antioxidant response, we measured the activities of key antioxidant enzymes. SOD and POD activities were initially induced but later declined under ABA treatment (Figure 2E,F). APX and CAT activities followed a similar trend, with significant suppression observed in ABA-treated petals by day 12 (Figure 2G,H). Moreover, transiently increased flower diameter on day 4, but no significant differences were detected at other time points, suggesting a brief promoting effect on flower expansion during early treatment (Figure S1). These results demonstrate that ABA accelerates membrane degradation and disrupts cellular redox balance in rose petals, whereas inhibiting ABA biosynthesis mitigates these detrimental effects.

### 3.3. ABA Treatment Inhibits RcMYB002 Gene Expression

**Figure 3:**
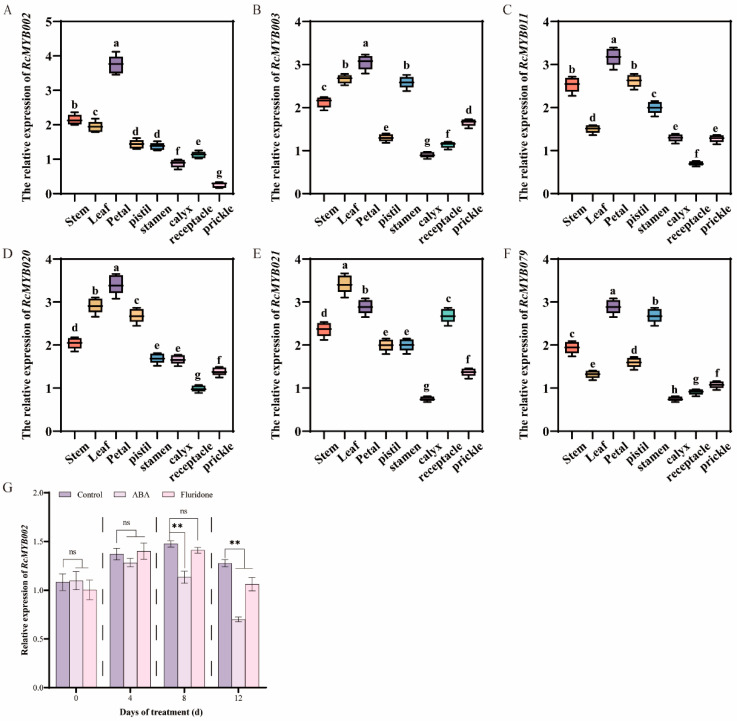
Abscisic acid treatment inhibits *RcMYB002* expression in rose petals. (**A**–**F**) Quantitative RT-PCR analysis showing the tissue-specific expression patterns of six MYB-related genes in various rose organs. (**G**) Temporal expression dynamics of *RcMYB002* in rose petals following treatment with Control (deionized water), ABA (50 μM), or fluridone (50 μM) at the indicated time points. Asterisks indicate significant differences between the treatment and Control groups within the same time point. ** *p* < 0.01 (Student’s *t*-test); ‘ns’ indicates no significant difference. Different letters denote significant differences among tissues (one-way ANOVA followed by Duncan’s multiple range test, *p* < 0.05). All experiments were performed with at least three biological replicates. Data are presented as mean ± SE.

To investigate whether ABA regulates MYB transcription factors during rose petal senescence, we selected six MYB-related genes highly enriched in abscisic acid response elements (ABREs) in their promoter regions as candidate genes, according to the promoter cis-element analysis by Zhu et al. [23]. Expression profiling of these genes across different tissues revealed that *RcMYB002* was preferentially expressed in petals, with significantly higher transcript levels compared to stems, leaves, pistils, stamens, sepals, receptacles, and prickles (Figure 3A). Moreover, among the six candidates, *RcMYB002* exhibited the highest expression in petals (Figure 3A–F), suggesting a potential role in petal physiology. Consequently, *RcMYB002* was selected for further functional characterization. To determine whether ABA influences *RcMYB002* expression, we examined its transcript levels in response to ABA treatment. Time-course analysis showed that ABA treatment caused a gradual decrease in *RcMYB002* expression over time (Figure 3G). In contrast, expression remained largely unchanged in fluridone-treated petals relative to the control. These findings indicate that ABA negatively regulates *RcMYB002* expression, implicating this MYB transcription factor in the ABA-mediated regulation of rose petal senescence.

### 3.4. RcMYB002 Inhibits the Aging of Rose Petals

**Figure 4:**
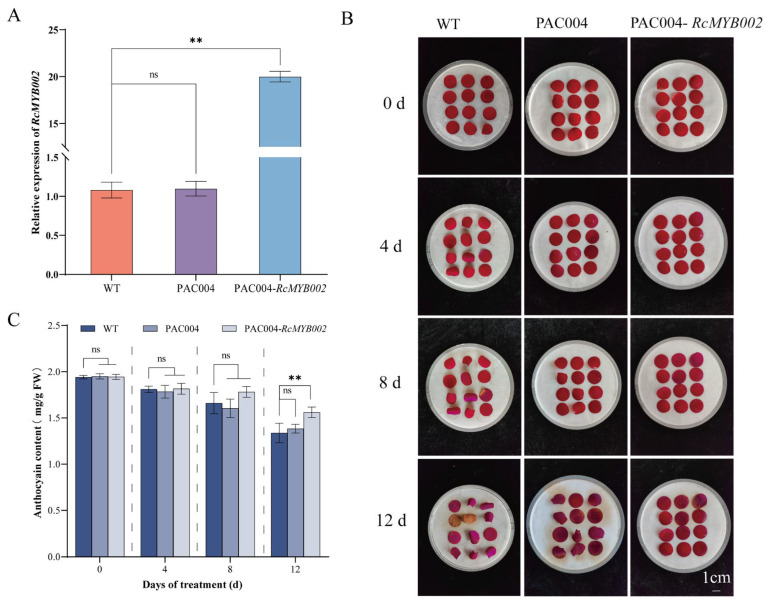
Overexpression of *RcMYB002* delays petal senescence in rose. (**A**) Quantitative RT-PCR analysis confirming elevated *RcMYB002* transcript levels in transiently overexpressing petals relative to control. (**B**) Representative images showing the senescence progression of petals isolated from wild-type (WT), empty vector control (pAC004), and *RcMYB002*-overexpressing plants at the designated time points. (**C**) Quantification of anthocyanin content in WT, pAC004 control, and *RcMYB002*-overexpressing petals. Values represent mean ± SE from three independent biological replicates (*n* = 3). Statistical significance was determined by Student’s *t*-test (** *p* < 0.01 compared with WT within the same time point); ‘ns’ indicates no statistically significant difference.

**Figure 5:**
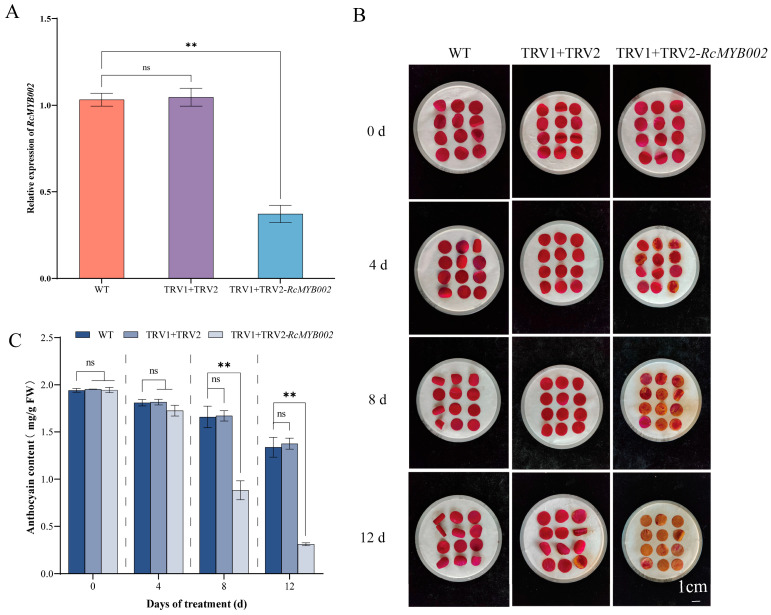
Virus-induced gene silencing of *RcMYB002* promotes petal senescence in rose. (**A**) Quantitative RT-PCR analysis confirming reduced *RcMYB002* transcript levels in VIGS-treated petals relative to control. (**B**) Representative images showing the senescence progression of petals isolated from wild-type (WT), empty vector control (TRV1 + TRV2), and *RcMYB002*-silenced plants at the designated time points. (**C**) Quantification of anthocyanin content in WT, TRV1 + TRV2 control, and *RcMYB002*-silenced petals. Values represent mean ± SE from three independent biological replicates (*n* = 3). Statistical significance was determined by Student’s *t*-test (** *p* < 0.01 compared with WT within the same time point); ‘ns’ indicates no statistically significant difference.

To further investigate the functional role of *RcMYB002* in rose petal senescence, we performed overexpression and silencing assays. The efficiency of overexpression and silence was confirmed by qRT-PCR, with *RcMYB002* transcript levels significantly elevated in overexpression lines and markedly reduced in silenced petals (Figure 4A and Figure 5A). Phenotypic observations showed that petals overexpressing *RcMYB002* exhibited delayed senescence compared to WT and empty vector (PAC004) (Figure 4B). Conversely, silencing of *RcMYB002* accelerated petal wilting and color fading (Figure 5B). Consistently, anthocyanin content was higher in *RcMYB002*-overexpressed petals and lower in silenced petals relative to controls (Figure 4C and Figure 5C), further supporting the role of RcMYB002 in maintaining petal pigmentation and delaying senescence.

**Figure 6:**
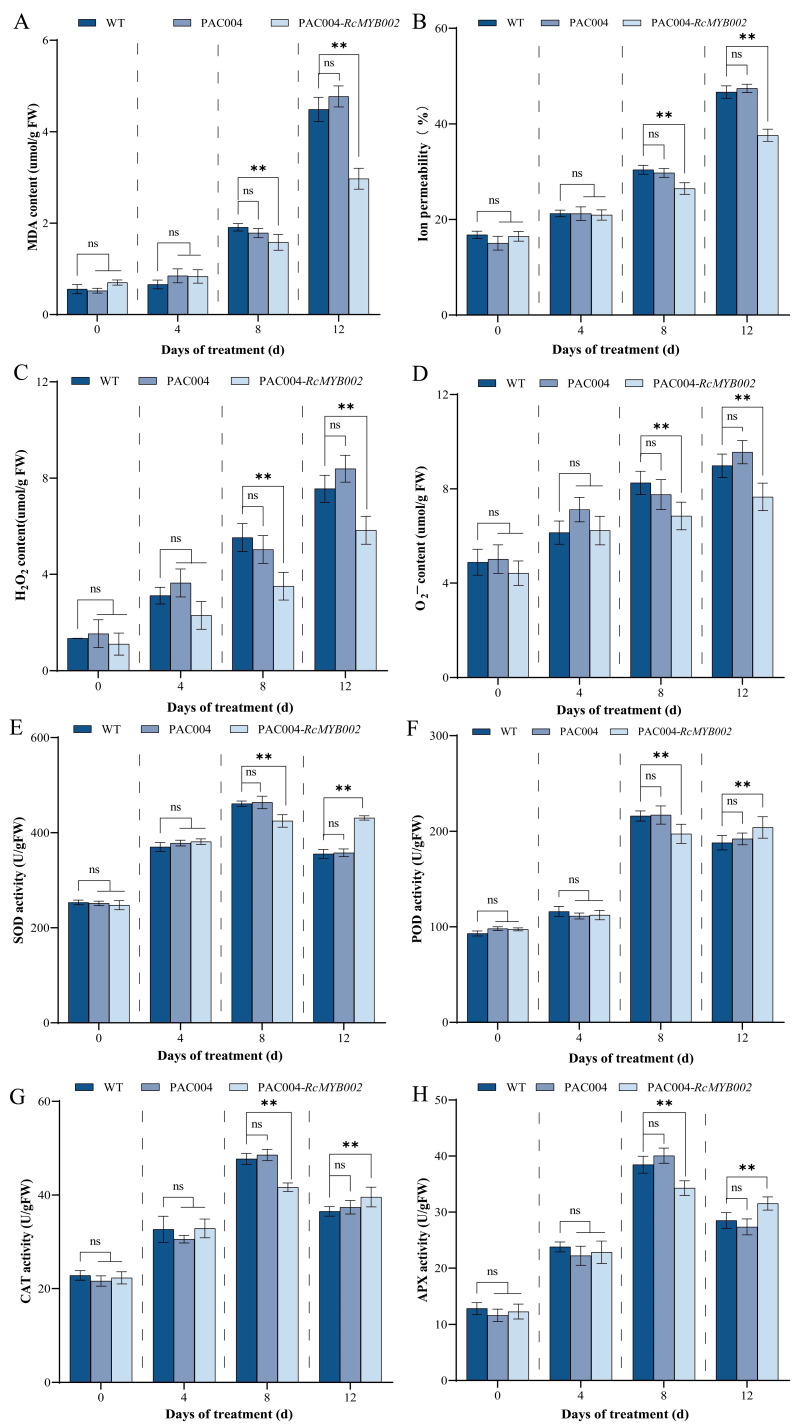
Physiological consequences of *RcMYB002* overexpression in rose petals. Comparative analysis of oxidative stress markers and antioxidant enzyme activities in wild-type (WT), empty vector control (pAC004), and *RcMYB002*-overexpressing petals. Parameters measured include: (**A**) malondialdehyde (MDA) content, an indicator of lipid peroxidation; (**B**) electrolyte leakage rate, reflecting membrane permeability; (**C**) hydrogen peroxide H_2_O_2_ content; (**D**) superoxide anion (O_2_^−^) content; and activities of antioxidant enzymes, (**E**) superoxide dismutase (SOD), (**F**) peroxidase (POD), (**G**) ascorbate peroxidase (APX), and (**H**) catalase (CAT). All measurements were performed at the indicated time points post-treatment. Values represent mean ± SE from three independent biological replicates (*n* = 3). Asterisks denote statistically significant differences compared with WT within the same time point as determined by Student’s *t*-test (** *p* < 0.01); ‘ns’ indicates no significant difference.

We further examined the effects of RcMYB002 modulation on oxidative stress and membrane integrity. Overexpression of *RcMYB002* significantly reduced MDA content and electrolyte leakage at 8 and 12 days of treatment, indicating attenuated membrane damage (Figure 6A,B). Moreover, overexpression lines exhibited lower levels of ROS (H_2_O_2_ and O_2_^−^) at 8 and 12 days (Figure 6C,D). Regarding antioxidant enzyme activities, overexpression of RcMYB002 led to significantly lower activities of SOD, POD, CAT, and APX at 8 days of treatment compared with control lines. At 12 days, the activities of SOD, POD, CAT, and APX in overexpression lines were significantly higher than those in controls, although the magnitude of increase varied among enzymes (Figure 6E–H). No significant differences in these parameters were observed at 0 or 4 days of treatment, highlighting a time-dependent enhancement of antioxidant defense capacity by RcMYB002.

**Figure 7:**
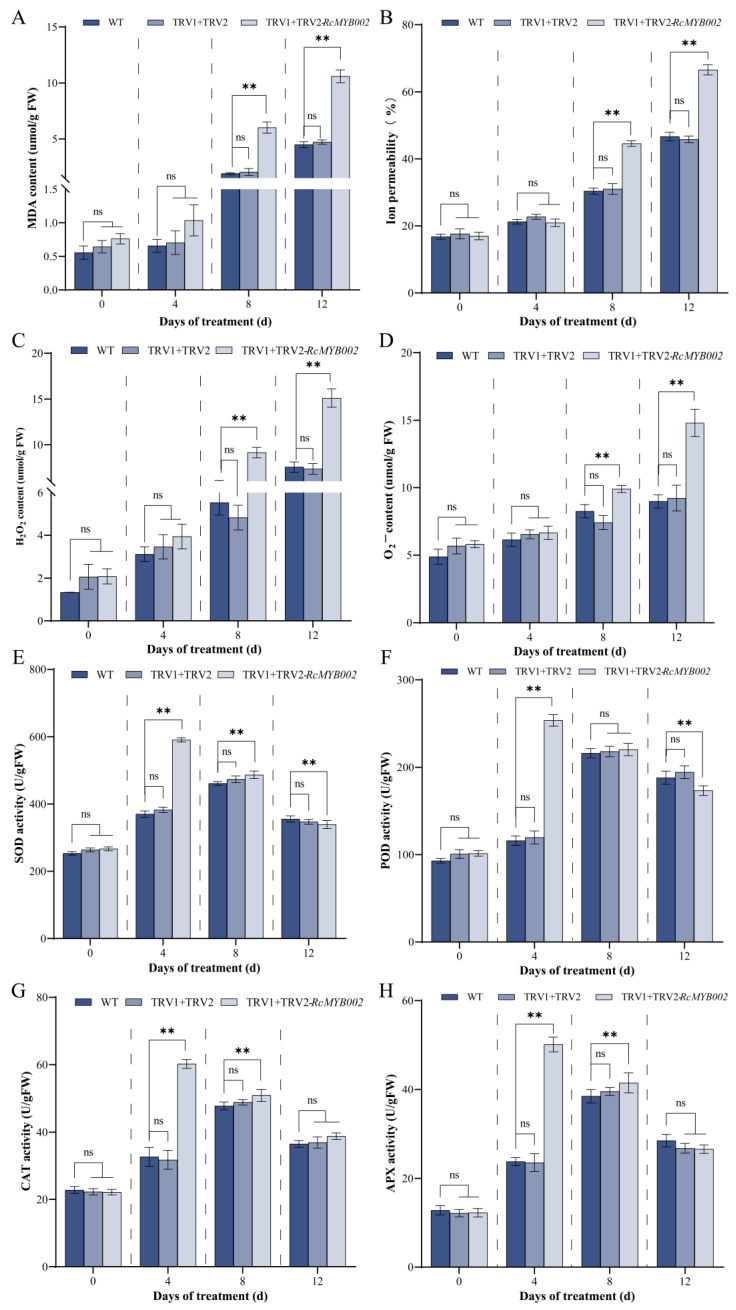
Physiological consequences of *RcMYB002* silencing in rose petals. Comparative analysis of oxidative stress markers and antioxidant enzyme activities in wild-type (WT), empty vector control (TRV1 + TRV2), and *RcMYB002*-silenced petals. Parameters measured include: (**A**) malondialdehyde (MDA) content, an indicator of lipid peroxidation; (**B**) electrolyte leakage rate, reflecting membrane permeability; (**C**) hydrogen peroxide H_2_O_2_ content; (**D**) superoxide anion (O_2_^−^) content; and activities of antioxidant enzymes: (**E**) superoxide dismutase (SOD), (**F**) peroxidase (POD), (**G**) ascorbate peroxidase (APX), and (**H**) catalase (CAT). All measurements were performed at the indicated time points post-treatment. Values represent mean ± SE from three independent biological replicates (*n* = 3). Asterisks denote statistically significant differences compared with WT within the same time point as determined by Student’s *t*-test (** *p* < 0.01); ‘ns’ indicates no significant difference.

In contrast, silencing of *RcMYB002* led to opposite effects in a time-dependent manner: at 8 and 12 days of treatment, MDA content and electrolyte leakage were significantly increased in silenced lines compared with controls (Figure 7A,B), accompanied by markedly elevated accumulation of H_2_O_2_ and O_2_^−^ (Figure 7C,D). Regarding antioxidant enzyme activities, silencing of *RcMYB002* resulted in significantly higher activities of SOD, POD, CAT, and APX at 4 days of treatment. At 8 days, the activities of SOD, APX, and CAT remained significantly higher in silenced lines, while POD activity showed no significant difference between groups. At 12 days, the activities of SOD and POD in silenced lines were significantly reduced, whereas CAT and APX activity exhibited no significant change compared with Control (Figure 7E–H). No significant differences in these parameters were detected at 0 days of treatment. These results suggest that RcMYB002 contributes to maintaining membrane stability and redox homeostasis, supporting its role as a negative regulator of petal senescence.

### 3.5. ABA-Induced Promotion of Petal Senescence Is Dependent on RcMYB002

**Figure 8:**
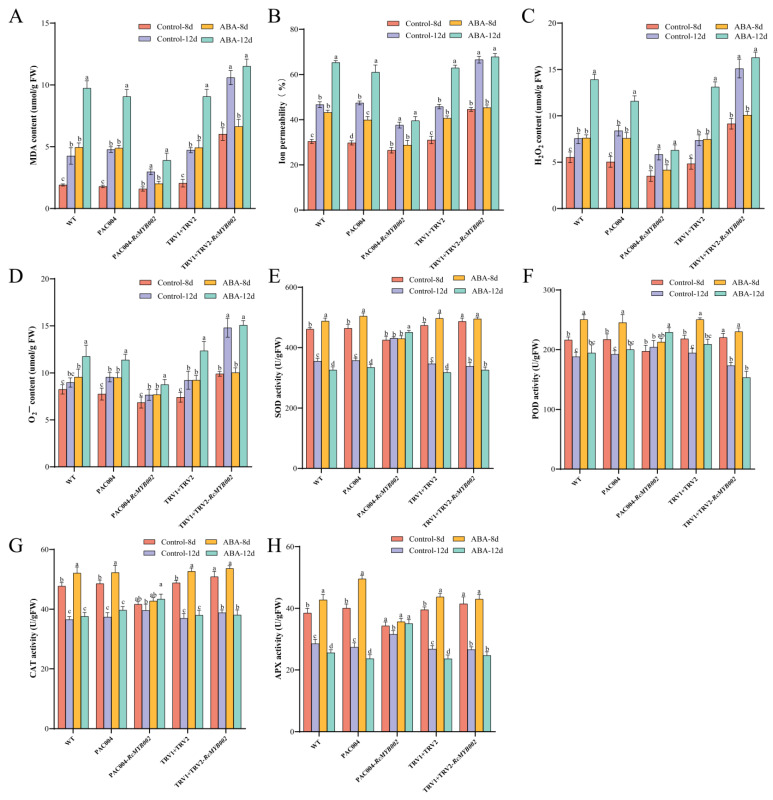
RcMYB002 mediates ABA-induced physiological responses in rose petals. Quantitative analysis of oxidative stress markers and antioxidant enzyme activities in wild-type (WT), *RcMYB002*-overexpressing (OE), and *RcMYB002*-silenced (VIGS) rose petals following treatment with ABA. Parameters assessed include: (**A**) malondialdehyde (MDA) content, (**B**) electrolyte leakage rate, (**C**) hydrogen peroxide H_2_O_2_ content, (**D**) superoxide anion (O_2_^−^) content, (**E**) superoxide dismutase (SOD) activity, (**F**) peroxidase (POD) activity, (**G**) ascorbate peroxidase (APX) activity, and (**H**) catalase (CAT) activity. All measurements were performed at the indicated time points. Values represent mean ± SE from three independent biological replicates (*n* = 3). Different lowercase letters denote statistically significant differences among treatment groups as determined by one-way analysis of variance (ANOVA) followed by Duncan’s multiple range test (*p* < 0.05).

To determine whether ABA-induced senescence is mediated through RcMYB002, we examined the responses of RcMYB002-overexpressing and silenced petals to ABA treatment. As shown in Figure 8, ABA treatment caused pronounced membrane damage in WT petals, as indicated by increased MDA content and electrolyte leakage. (Figure 8A,B). Similarly, ABA-induced ROS (H_2_O_2_ and O_2_^−^) was significantly alleviated in *RcMYB002*-overexpressing lines but aggravated in silenced lines (Figure 8C,D). Correspondingly, under ABA treatment, *RcMYB002*-overexpressing petals showed generally higher activities of key antioxidant enzymes such as SOD, POD, APX, and CAT, whereas these enzyme activities were further reduced in silenced petals relative to controls (Figure 8E–H). Notably, the activities of SOD and APX showed opposite trends at 8 d and 12 d of ABA treatment: at early senescence stages (8 d), ABA induced a transient upregulation of antioxidant capacity, whereas prolonged ABA treatment (12 d) accelerated petal senescence and resulted in reduced enzyme activity relative to the control. This dynamic response reflects the stage-dependent regulation of ABA on antioxidant metabolism during rose petal senescence. Thus, these results indicate that RcMYB002 functions to counteract ABA-induced oxidative stress and membrane damage, thereby delaying petal senescence.

**Figure 9:**
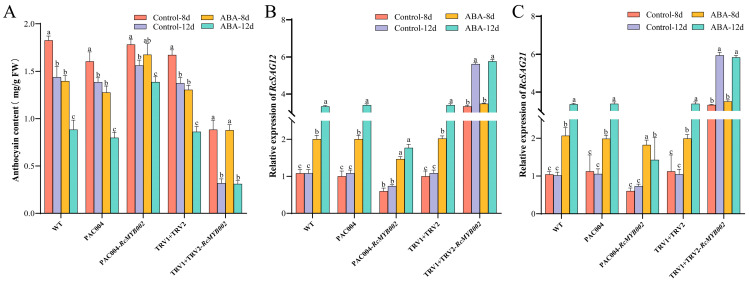
RcMYB002 modulates ABA-induced anthocyanin degradation and SAG expression. Quantitative analysis of (**A**) anthocyanin content, (**B**) *RcSAG12* transcript levels, and (**C**) *RcSAG21* transcript levels in wild-type (WT), *RcMYB002*-overexpressing (OE), and *RcMYB002*-silenced (VIGS) rose petals following ABA treatment. Expression levels were normalized to an internal control and are presented relative to WT. Values represent mean ± SE from three independent biological replicates (*n* = 3). Different lowercase letters denote statistically significant differences among treatment groups as determined by one-way analysis of variance (ANOVA) followed by Duncan’s multiple range test (*p* < 0.05).

Consistent with these oxidative stress responses, anthocyanin content declined more rapidly in ABA-treated *RcMYB002*-silenced petals, whereas it was better preserved in overexpression lines (Figure 9). Furthermore, ABA treatment significantly upregulated the expression of senescence-associated genes *SAG12* and *SAG21* in WT petals. This induction was suppressed in *RcMYB002*-overexpressing petals but further enhanced in silenced petals (Figure 9). We also analyzed the expression of ABA-related genes under the same conditions. As shown in Figure S2, key ABA synthesis gene *NCED1* and signaling pathway genes (*PYL4*, *PYL8*, *ABF*, *ABI1*) were induced by ABA in control petals. Notably, overexpression of *RcMYB002* attenuated this upregulation, while silencing resulted in more pronounced expression of these ABA-responsive genes, indicating that RcMYB002 affects the expression of several ABA-responsive genes during petal senescence. Collectively, these results demonstrate that ABA-induced membrane damage, ROS accumulation, antioxidant system imbalance, and senescence-related gene expression are all significantly influenced by RcMYB002. Overexpression of *RcMYB002* antagonizes ABA-accelerated senescence, while its silencing enhances petal sensitivity to ABA, positioning RcMYB002 as a key node in the regulatory network governing petal longevity.

### 3.6. The Transcriptional Regulation of Aging-Related Genes SAG12 and SAG21 by RcMYB002

**Figure 10:**
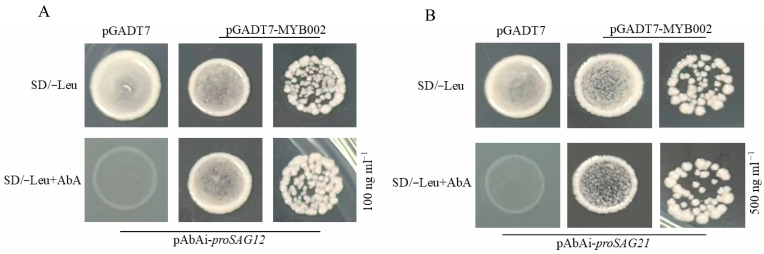
RcMYB002 directly targets the promoters of senescence-associated genes *SAG12* and *SAG21*. Yeast one-hybrid (Y1H) assays were performed to examine the transcriptional regulatory activity of RcMYB002 on the promoters of *SAG12* and *SAG21*. (**A**) The promoter fragment of *SAG12* was cloned into the pBait-AbAi reporter vector, and the coding sequence of RcMYB002 was fused to the GAL4 activation domain in the pGADT7 prey vector. (**B**) Similarly, the *SAG21* promoter fragment was inserted into pBait-AbAi, and RcMYB002 was expressed as a pGADT7 fusion protein. The resulting constructs were co-transformed into the Y1H Gold yeast strain. Positive protein-DNA interactions were evidenced by yeast cell growth on selective medium supplemented with aureobasidin A (AbA), as shown in the panels. Empty pGADT7 vector was used as a negative control.

To elucidate the molecular mechanism underlying RcMYB002-mediated regulation of petal senescence, we conducted Y1H assays to identify potential transcriptional targets. Given the altered expression patterns of senescence-associated genes (*SAG12* and *SAG21*) observed in *RcMYB002*-overexpressing and silenced petals (Figure 9B,C), we hypothesized that these SAGs might be regulated by RcMYB002. To verify this hypothesis, we generated yeast reporter strains harboring the promoter regions of *SAG12* and *SAG21*. Subsequent Y1H analysis demonstrated that the RcMYB002 protein interacts with the promoter sequences of both *SAG12* and *SAG21* (Figure 10A,B). These results indicate that RcMYB002 regulates petal senescence, at least in part, by affecting the expression of key senescence-associated genes.

## 4. Discussion

Senescence represents the terminal phase of plant development, manifesting at cellular, tissue, and organ levels, with far-reaching agronomic implications. This process substantially restricts crop yield and biomass accumulation while constituting a major contributor of postharvest losses in fruits, vegetables, and ornamental commodities during storage and distribution [39]. The initiation and progression of senescence are governed by both environmental stimuli and endogenous developmental programs. Various biotic and abiotic stressors, including pathogen challenge, drought, nutrient limitation, etiolation, and temperature extremes, can precipitate premature senescence [40]. Concurrently, senescence proceeds as an age-dependent process orchestrated by a sophisticated phytohormonal network. Senescence-promoting phytohormones, namely ABA and ethylene, accelerate cellular deterioration, whereas gibberellins and cytokinins antagonize these effects, collectively modulating senescence dynamics through intricate regulatory crosstalk [41]. Among these hormonal regulators, ABA is firmly established as a natural accelerator of flower senescence. Corroborating this paradigm, our experimental data demonstrates that exogenous ABA treatment significantly accelerates petal senescence in rose (Figure 1). These observations align with previous reports documenting ABA-induced senescence-associated events, including membrane permeability loss and lipid peroxidation in daylily flowers [39] and premature accumulation of senescence-associated transcripts in daffodil (*Narcissus pseudonarcissus*) tepals [42]. Extending these findings, our results indicate that ABA accelerates rose petal senescence by enhancing oxidative stress and disrupting membrane integrity (Figure 2). Collectively, these results underscore the efficacy of exogenous ABA in modulating floral senescence dynamics. From an applied perspective, targeted hormonal manipulation during floral development may enhance flexibility of harvest scheduling, thereby furnishing a theoretical foundation and practical framework for growers to align harvesting and marketing strategies with dynamic market demands.

The commercial value of cut rose flowers is largely determined by the ornamental quality of fully opened blooms, which relies on fully expanded petals and an extended vase life. Consequently, the physiological mechanisms underlying rose flower senescence have remained an economically important research topic for several decades. Petal senescence is a highly regulated developmental process modulated by both endogenous signals and environmental stimuli, and its precise regulation is essential for maintaining the aesthetic and commercial value of cut flowers [43]. Within the phytohormonal network, ABA is well established as a key promoter of both leaf and flower senescence [37,44]. In alignment with previous investigations in diverse ornamental species such as lily [45], carnation [6], and tulip [46,47], our findings confirm that exogenous ABA significantly accelerates petal wilting, promotes anthocyanin degradation, and induces the expression of the senescence-associated genes *SAG12* and *SAG21* in rose petals (Figure 1). These observations establish a strong correlation between endogenous ABA levels and the progression of petal senescence in this economically valuable ornamental species. Mechanistically, we provide evidence that ABA treatment induces marked membrane lipid peroxidation, elevates electrolyte leakage, and disrupts the activities of key antioxidant enzymes in rose petals (Figure 2), all characteristic manifestations of oxidative damage [48]. This conclusion is further supported by previous reports of ABA-induced oxidative stress in marigold [44]. These results collectively demonstrate that ABA plays a crucial role in promoting rose petal senescence by inducing oxidative damage and regulating the expression of senescence-associated genes, thereby laying a physiological foundation for further exploring the molecular regulatory network underlying ABA-mediated petal senescence. It should be noted that, when examining the results under different ABA treatment durations, the trends of certain physiological indicators appear partially inconsistent with the overall conclusion of ABA-promoted senescence. These apparent discrepancies are mainly caused by the stage- and time-dependent effects of ABA during rose petal senescence. In the early phase of ABA treatment, petals tend to activate a stress-protective response to cope with exogenous stimuli, which may lead to transient changes in antioxidant enzyme activities that are not fully consistent with the senescence-promoting trend. As ABA treatment continues, its senescence-accelerating effect gradually dominates, accelerating petal senescence and resulting in opposite trends in some physiological parameters. Such dynamic and stage-dependent responses are normal physiological characteristics during plant senescence and are not caused by experimental errors or measurement inaccuracies.

MYB transcription factors constitute one of the largest and most functionally diverse gene families in plants, with well-documented roles in regulating abiotic stress responses, secondary metabolic pathways, senescence, and multiple developmental processes [15]. In the model species *Arabidopsis thaliana*, only a limited subset of MYB factors (including MYBL48, MYBH49, and MYB250) have been functionally associated with the regulation of plant senescence [49]. Within the ornamental crop rose, previous investigations have demonstrated that *RhMYB108* transcripts accumulate preferentially in petals relative to other organs such as roots, stems, and leaves [17]. Extending these observations, our tissue-specific expression profiling revealed that *RcMYB002* is predominantly expressed in petals, with significantly lower transcript abundance detected in vegetative tissues (stems and leaves) and other floral organs (pistils, stamens, and sepals) (Figure 3). This expression pattern corroborates earlier findings implicating petal-predominant MYB factors in the regulation of pigment biosynthesis and senescence in rose [17]. Through complementary gain- and loss-of-function approaches employing transient overexpression and VIGS, our study furnishes direct functional evidence establishing RcMYB002 as a negative regulator of petal senescence in rose. Ectopic expression of RcMYB002 significantly delayed senescence progression, preserved anthocyanin accumulation, and bolstered cellular antioxidant capacity, whereas targeted silencing of this gene accelerated senescence and potentiated ABA-induced oxidative damage (Figure 4, Figure 5, Figure 6 and Figure 7). These findings resonate with previous reports demonstrating that suppression of *RhMYB108* alters the expression of multiple senescence-associated genes (SAGs)—including *RhNAC029*, *RhNAC053*, *RhNAC092*, *RhSAG12*, and *RhSAG11*—thereby attenuating ethylene- and jasmonic acid-induced petal senescence [17]. Collectively, this investigation establishes RcMYB002 as a negative regulator of rose petal senescence functionally integrated with anthocyanin metabolism, antioxidant defense systems, and ABA signaling pathways. These findings not only expand the conceptual framework of MYB-mediated transcriptional regulation during petal senescence but also furnish valuable genetic resources and theoretical foundations for deciphering the molecular mechanisms governing rose petal longevity and for developing biotechnological strategies to extend the vase life of commercially important cut flowers.

MYB proteins are classified into R1-MYB, R2R3-MYB, R3-MYB, and R4-MYB subfamilies based on the number of conserved MYB repeats, and they participate in diverse biological processes including plant growth, hormone signal transduction, abiotic stress and disease resistance, and secondary metabolism. In this study, we provide mechanistic insights into how RcMYB002 modulates ABA-induced petal senescence. Under ABA treatment, petals overexpressing *RcMYB002* exhibited reduced membrane damage, lower ROS accumulation, and higher antioxidant enzyme activities compared to WT and gene-silenced lines (Figure 8 and Figure 9). Moreover, Y1H assays demonstrated that the RcMYB002 protein interacts with the promoter regions of senescence-associated genes *SAG12* and *SAG21* (Figure 10), both of which exhibit strict senescence-specific expression patterns and are widely used as molecular markers for senescence [50,51]. Consistent with our findings, recent studies in rice have found that MYB transcription factors can directly bind to the promoters of senescence-related genes, thereby regulating the leaf aging process [52,53]. Based on these results, we propose a working model in which ABA promotes petal senescence by suppressing the expression of *RcMYB002*. The down-regulation of *RcMYB002* reduces the inhibitory effect on the core senescence associated genes *SAG12* and *SAG21*, thereby activating a cascade of senescence-related cellular events, including oxidative stress, membrane damage, and pigment degradation. Conversely, enhanced RcMYB02 activity reinforces antioxidant defense systems, improves membrane stability, and represses the expression of *SAG*s, effectively delaying petal senescence. These findings suggest that modulation of RcMYB02 via ABA application represents a promising strategy for manipulating the senescence program in rose. Our study provides a clear framework for the relationship between ABA signaling and MYB-mediated transcriptional regulation in floral senescence and identifies RcMYB002 as a key regulatory node in this network.

## 5. Conclusions

The present study establishes that ABA functions as a positive regulator of petal senescence in rose, while RcMYB002 antagonizes this process. RcMYB002 interacts with the promoter regions of the senescence-associated marker genes *SAG12* and *SAG21* in yeast one-hybrid (Y1H) assays, consistent with a role in their transcriptional regulation. This regulatory effect of RcMYB002 consequently contributes to the preservation of cellular redox balance and membrane integrity during rose petal senescence. These findings significantly advance our mechanistic understanding of the regulatory interplay between phytohormonal signals and transcription factors in orchestrating floral senescence. Moreover, they identify RcMYB002 as a promising genetic target and provide a theoretical framework for biotechnological strategies aimed at extending the postharvest longevity of commercially valuable cut roses.

## Data Availability

All data, tables, and figures in this manuscript are original and contained within the article and Supplementary Materials. Gene Data are derived from public domain resources.

## Supplementary Materials

The following supporting information can be downloaded at https://www.mdpi.com/article/10.3390/antiox15040415/s1, Figure S1: The effect of ABA treatment on the flower stems of cut rose plant; Figure S2: Expression levels of ABA-related genes in RcMYB002 overexpression and silencing plants treated with ABA; Table S1: Primer sequences designed for qRT-PCR; Table S2: Primer sequences designed for Virus-induced gene silencing and overexpression; Table S3: Primer sequences designed for yeast one-hybrid (Y1H) assay.
