# Supplementary material for: Abscisic Acid Promotes Petal Senescence in Rose by Regulating RcMYB002
Source: Antioxidants (Basel). 2026 Mar 26;15(4):415. doi: 10.3390/antiox15040415 (PMC13113399; doi:10.3390/antiox15040415)
Supplement: Supplementary file 1 [file antioxidants-15-00415-s001.zip › antioxidants-4195614-supplementary.pdf]

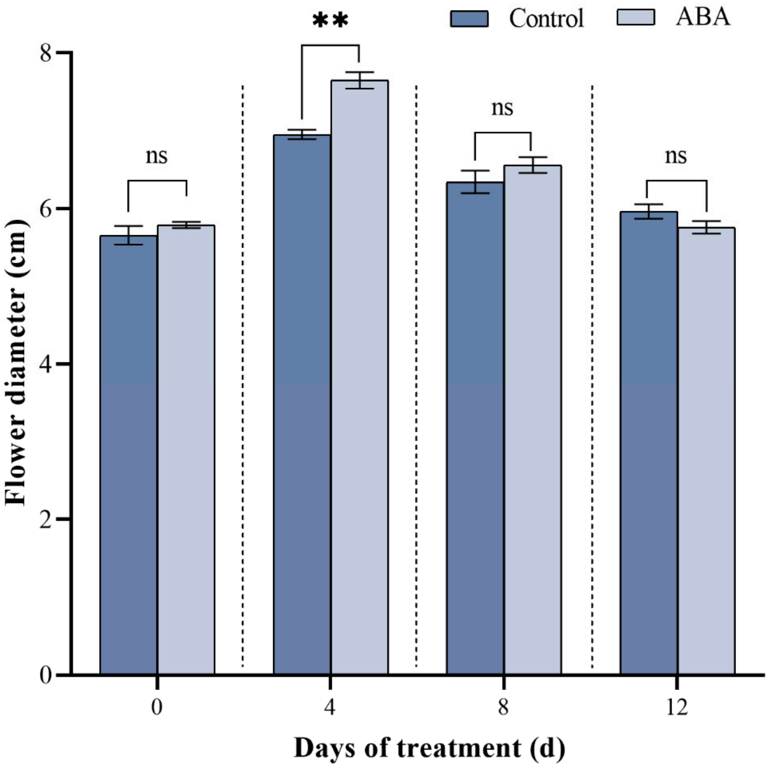

Figure S1. The effect of ABA treatment on the flower stems of cut rose plant. Data represent the mean ± SE of three biological replicates (n = 3). Asterisks indicate the presence of significant differences analyzed by Student's t-test (**P < 0.01). 'ns' indicates no significant differences compared with the Control.

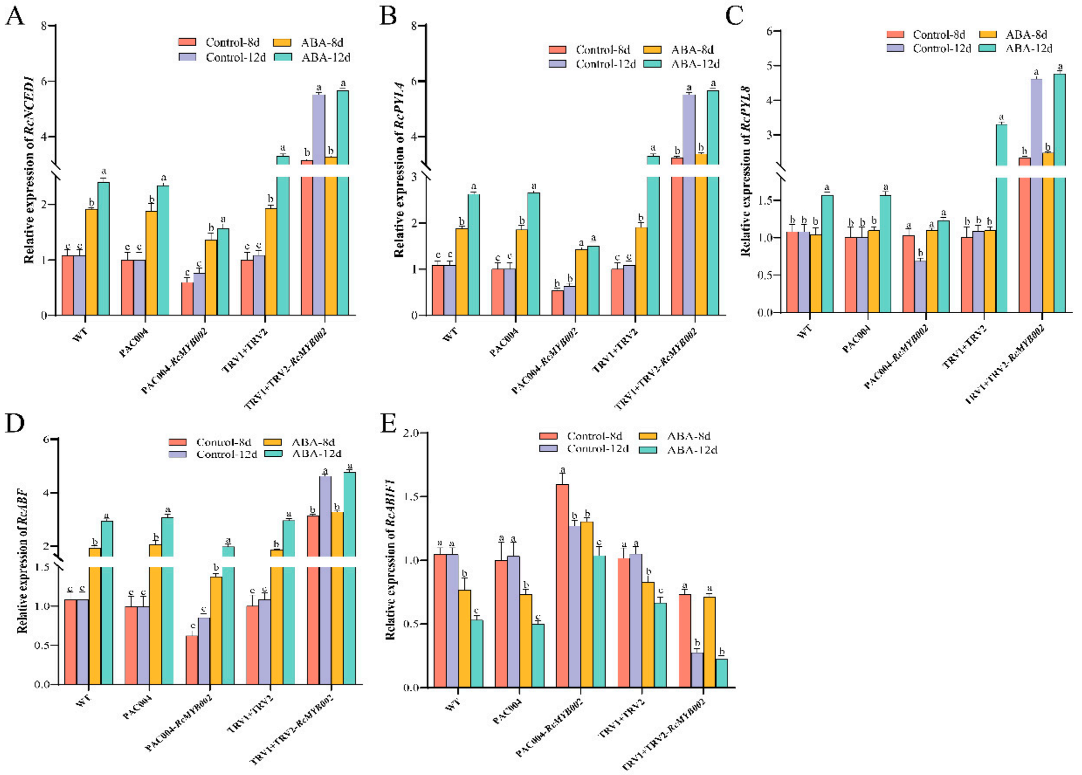

Figure S2. Expression levels of ABA-related genes in *RcMYB002* overexpression and silencing plants treated with ABA. (A-E) RT-qPCR analysis of ABA-related gene expression levels under ABA treatment. Values (means ± standard deviations) were calculated from three independent replicates. Different letters indicate significant differences compared with the Control (Student's *t*-test, $P<0.01$).

Table S1. Primer sequences designed for qRT-PCR.

Gene name	Accession number	Foward primer (5’-3’)	Reverse primer (5’-3’)
RcSAG12	XM_024301607	AGCGGAGAAGCCTTTCAGTC	CAGCATGGTTCAGGCTGGTA
RcSAG21	LOC112171950	GGATCTTCCAGAGATTCTCTCTGAAACCATCTTCGCA	CTGCCGTTCGACGATTTCATTTCTAAGAATATAAGTTTAATTTTATTGC
RcMYB002	rna-XM_040516774.1	CCTTTAGTCCTGCTGCTGTTGGG	CGTGTATGGTTTCCTCACCTTGGG
RcMYB003	rna-XM_024333184.2	CCTTTAGTCCTGCTGCTGTTGGG	CGTGTATGGTTTCCTCACCTTGGG
RcMYB011	rna-XM_024307506.2	ACAAGGGAGTGGGATGGAGTGAG	TGACTGGCGACCTGGGTTGG
RcMYB020	rna-XM_040506100.1	AATGCTGGTTGCCGTACATCGTC	CTCGCCACTTTCCCTTGCTCTG
RcMYB021	rna-XM_024300926.2	AATGCTGGTTGCCGTACATCGTC	CTCGCCACTTTCCCTTGCTCTG
RcMYB079	rna-XM_024331915.2	CGTCCAAAGAAGCAGGCAATTCG	TCGCCCTTTCATTGTCAGACTTGG

Table S2. Primer sequences designed for Virus-induced gene silencing and overexpression.

Gene name	Foward primer (5'-3')	Reverse primer (5'-3')
TRV-MYB002	AAGGTTACCGAATTCTCTAGAAAGGTTGCTCGTGACCCAGA	CGTGAGCTCGGTACCGGATCCACCAGCAACCAATGAAACAGGG
HA-SAG12	GGCGCGCCGATATCGTCGACATGGCGTCTCCAGCTATGGCAC	CGTATGGGTAGGTACCCTAACAAAGGTGGACTCTTTTC

Table S3. Primer sequences designed for yeast one-hybrid (Y1H) assay.

Gene name	Foward primer (5'-3')	Reverse primer (5'-3')
AD-MYB002	GTACCAGATTACGCTCATATGATGGCGTCTCCAGCTATGGC	ACGATTCATCTGCAGCTCGAGCTAACAAAGGTGGACTCTTTTCTGAT
Pabi-SAG12	CTTGAATTCGAGCTCGGTACCTGGCCAAACAGCCAGACTTG	ATACAGAGCACATGCCTCGAGGACCCTAGAAAACTTCTTAGTGGAGAT
Pabi-SAG21	CTTGAATTCGAGCTCGGTACCCCACACCATTCATTCATGTTCATT	ATACAGAGCACATGCCTCGAGGCGGTGGGAGAGTTCTTTGG
